# Chemical characterization and nutritional potential of a Costa Rican edible cricket (*Gryllus* sp.) for human consumption

**DOI:** 10.3389/fnut.2026.1816151

**Published:** 2026-07-29

**Authors:** Ana María Quirós, Milieth Arias-Leitón, Sergio Jansen-González, Graciela Artavia, Luis Ricardo Murillo-Hiller, Viky Calero, Oscar Abel Sánchez-Velázquez, Oscar Acosta

**Affiliations:** 1 Department of Food Engineering, Guanacaste Campus, University of Costa Rica, Liberia, Costa Rica; 2 National Center of Food Science and Technology (CITA), University of Costa Rica, San José, Costa Rica; 3 School of Biology and Research Center for Biodiversity and Tropical Ecology (CIBET), University of Costa Rica, San José, Costa Rica; 4 Department of Ecotourism, Guanacaste Campus, University of Costa Rica, Liberia, Costa Rica; 5 School of Food Science & Nutrition, University of Leeds, Leeds, United Kingdom

**Keywords:** amino acid profile, cardiometabolic indices, edible insects, fatty acid composition, protein quality, sustainable protein

## Abstract

This study characterized the morphology, proximate composition, micronutrient profile, amino acid distribution, and fatty acid composition of a *Gryllus* sp. powder to evaluate its nutritional quality. Morphological examination assigned the specimens to the genus *Gryllus* while suggesting possible divergence from currently described species. Protein content calculated using chitin-corrected nitrogen reached 61.48 g/100 g dry matter, providing an improved estimate of true protein content. Total fat was 11.65 g/100 g, while ash 5.01 g/100 g and dietary fiber 7.43 g/100 g, indicating limited discriminatory power of proximate composition alone. The mineral profile showed elevated macro-elements, particularly K (1,037.50 mg/100 g), P (915.00 mg/100 g), and Ca (313.75 mg/100 g), while *α*-tocopherol (23.80 mg/100 g) and vitamin C (11.64 mg/100 g) revealed a distinctive micronutrient pattern. The essential amino acid profile yielded an EAA/TAA ratio of 56.51%, supporting favorable protein quality; however, Lys (34.78 mg/g protein) was identified as the first limiting amino acid relative to FAO reference patterns, differing from limitation patterns reported for other *Gryllus* species. The lipid fraction was dominated by linoleic (34.29%) and oleic acids (30.66%), resulting in favorable cardiometabolic indices characterized by low atherogenicity (0.63) and thrombogenicity (0.53). Overall, the nutritional profile confirms the potential of *Gryllus* sp. as a high-quality protein source with favorable lipid quality indices and a potentially favorable cardiometabolic fatty acid profile. Comprehensive nutritional profiling therefore represents a useful complementary approach for the characterization of edible cricket materials.

## 1 Introduction

Edible insects have emerged as alternative protein sources with high nutritional value and low environmental impact, positioning them as a viable solution to current challenges in global food systems (1–3). Studies have reported wide ranges of protein, lipid, fiber, and ash contents on a dry matter basis, highlighting their potential as functional food ingredients (2, 4, 5). In addition, their high feed conversion efficiency and reproductive capacity have further stimulated scientific interest in this group. Globally, Coleoptera, Lepidoptera, Hymenoptera, Orthoptera, and Hemiptera account for most edible insect consumption (6, 7).

Nevertheless, nutritional research remains largely focused on a limited number of commercially farmed and officially authorized species for human consumption, such as *Tenebrio molitor*, *Locusta migratoria*, *Alphitobius diaperinus*, and *Acheta domesticus* (8–11). This concentration restricts current knowledge of the true diversity of edible insects with nutritional potential. Considering the projected growth of the global population (12), it is necessary to expand research toward a broader diversity of edible species. Such diversification could contribute to food security through the development of nutritious and affordable protein sources (13).

Within this context, orthopterans, particularly crickets, stand out as one of the most widely consumed groups of edible insects worldwide and in Costa Rica (14–16). Commonly reported edible cricket species include *Brachytrupes membranaceus*, *Gryllus assimilis*, *Gryllus bimaculatus*, *Gryllotalpa orientalis*, and *Acheta domesticus* (4, 10, 17–20). Notably, other edible cricket species within the family Gryllidae, particularly those belonging to the genus *Gryllus*, remain poorly studied.

The nutritional composition of crickets can vary even within the same species, depending on factors such as developmental stage, habitat, climate, sex, and feeding substrate (4, 10, 15, 21, 22). Reported values for different cricket species range of 18.6–71.1 g/100 g dry weight for protein, 6.0–34.0 g/100 g for lipids, 0.5–13.3 g/100 g for fiber, 2.5–47.2 g/100 g for carbohydrates, and 3.0–12.6 g/100 g dry weight for ash (15). These variations highlight the nutritional diversity among cricket species worldwide and emphasize the need for further research, particularly in tropical regions with high biodiversity.

In Costa Rica, generating scientific information on local edible cricket species, such as *Gryllus* sp., is essential to support their chemical characterization and potential future incorporation as a sustainable food alternative for human consumption. In this context, the application of conventional nutritional quality assessment techniques, including the determination of moisture, ash, dietary fiber, total fat, chitin, carbohydrates, vitamins, minerals, and protein content, allows the establishment of a reliable nutritional profile comparable to that of other edible cricket species and orthopterans reported in the literature (15).

Although, despite the traditional consumption of crickets and other edible insects in Costa Rica, many locally utilized populations remain poorly characterized from a morphological and taxonomic standpoint, limiting robust species-level identification. Beyond their nutritional relevance, compositional parameters may also provide insight into species-specific metabolic traits, as protein, lipid, and mineral deposition are partially regulated by intrinsic physiological and genetic factors (23). Therefore, comprehensive compositional profiling, when interpreted alongside morphological evidence, may provide complementary information for comparing nutritional variation among *Gryllus* populations. Despite this, nutritional composition alone should not be considered evidence for species delimitation.

Beyond proximate composition and micronutrient determination, assessing complementary nutritional quality indicators is essential for a comprehensive evaluation. Protein quality is particularly important, as it depends not only on total protein content but also on amino acid composition and its adequacy relative to human requirements. Comparison with international reference patterns (24) enables the identification of limiting amino acids and the estimation of biological adequacy. Accordingly, indices such as the Amino Acid Score (AAS), Essential Amino Acid Index (EAAI), and predicted Biological Value (BV) provide insight into the metabolic utilization potential of the protein fraction (25). Similarly, fatty acid profiling and the calculation of lipid quality indices, including the Atherogenicity Index (AI) and Thrombogenicity Index (TI), help estimate the potential impact of dietary lipids on cardiovascular health (26, 27). The integration of these indicators strengthens the evaluation of the nutritional value of *Gryllus* species and supports its consideration as a sustainable protein source for human diets.

The objective of this study was to conduct a comprehensive chemical and nutritional characterization of an edible cricket species from the genus *Gryllus* (*Gryllus* sp.) from Costa Rica, using conventional analytical methods, to generate comparable scientific data to support its evaluation as a sustainable protein source and to contribute to the diversification of edible insects and alternative protein sources for human nutrition.

## 2 Materials and methods

### 2.1 Insect rearing and voucher specimens

Adults of *Gryllus* sp. were obtained from a local farmer. Ten females and ten males were used to start the breeding system, from this stock another two generations were bred to obtain enough biomass for the physicochemical analyses. Four reproduction chambers were established using 45 L transparent plastic storage boxes, each containing an independent group of *Gryllus* sp. (*n* = 4). Shelter was provided with pilled, folded carton eggs inside the boxes, egg laying substrate consisted of 90–100 g of softly compacted, moistened coconut fiber contained in 10 cm diameter × 10 cm high plastic pots covered with 0.2 mm metallic mesh to allow females access with their ovipositor but not with their mouthparts, avoiding egg cannibalism. Food and water were offered using separate petri dishes (10 cm diameter × 1.5 cm high). Food consisted of grinded dog food (Member’s Selection Fun and Faithful) and laying hen feed mixed in equal proportions. A 25 W incandescent light bulb fixed in the lateral interior of each box was used as light and heat source, turned in a 12 h photoperiod. Each storage box was covered with organdy at the top to allow ventilation. Eggs laid during a 4–5 days period in the coconut fiber pots was taken to separate plastic boxes and let the nymphs emerge; the feeding protocols mentioned above were used for the development of the insects. The same setup for the reproductive chambers was used to raise the nymphs until reaching harvesting size, around 3–4 cm body length. Digital images of these specimens were taken using a Canon EOS Rebel T3i DSLR camera with Canon MP-E 65 mm f/2.8 1-5X macro lens attached.

### 2.2 Sample treatment

All samples were initially stored at −18 °C. For chemical analysis, first the samples were first freeze–dryed (Benchtop 4.5 L, Labconco, FreeZone, Kansas City, MO, USA) and sieved to a particle size of 1 mm using a cyclone mill (Twister, Retsch, Haan, Germany) and subsequently stored at −80 °C in vacuum-sealed polyethylene bags until analysis.

### 2.3 Chemical reagents

Analytical standards as: ascorbic acid (catalog number PHR1008), *α*-tocopherol acetate, α-tocopherol, *β*-tocopherol, *δ*-tocopherol, and *γ*-tocopherol standards (catalog number W530066), Retinyl acetate (catalog number 46958), retinyl palmitate (catalog number 46959), ergocalciferol (catalog number PHR1238), cholecalciferol (catalog number PHR1237), pyridoxal 5′-phosphate hydrate (catalog number P9255), pyridoxine hydrochloride (catalog number P5669), folic acid (catalog number PHR1035), riboflavin (catalog number PHR1054), thiamine hydrochloride (catalog number PHR1037), cyanocobalamin (catalog number PHR1234), nicotinic acid (catalog number 72309) and niacinamide (catalog number 72340) were purchased from Millipore Sigma (Saint Louis, MO, USA). Catalyst Kjeltabs Cu/3.5 (catalog number 60046515) was purchased from FOSS Hilleroed, Denmark). Sulphuric acid (ACS reagent, 95–98%, catalog number 258105), hydrochloric acid (37%, ACS reagent, catalog number 258149), boric acid (catalog number 1.93409), sodium hydroxide (ACS reagent, ≥97.0%, catalog number 221465), metaphosphoric acid (ACS reagent, catalog number 239275), sodium sulfate (anhydrous, ACS reagent, powder, catalog number, 238597), sodium 1-heptanesulfonate (for ion pair chromatography, catalog number 1.18306), tris [2-carboxyethyl] phosphine hydrochloride (powder, catalog number C4706), 2,6-Di-tert-butyl-4-methylphenol (BHT ≥ 99.0% GC powder, catalog number B1378), chloroform (ACS reagent, catalog number 366919), 2-propanol (99.9% suitable for HPLC, catalog number 34863), petroleum ether (ACS reagent, catalog number 184519), methanol (MeOH, ≥99.9%, suitable for HPLC catalog number 646377) and total dietary fiber assay kit (catalog number TDF100A) were purchased from Millipore Sigma Saint Louis, MO, USA. And ultrapure water [type I, 0.055 μS cm − 1 at 25 °C, 5 μg L^−1^ TOC] was obtained using an A10 Milli-Q Advantage system and an Elix 35 system (Merck KgaA, Darmstadt, Germany). The reagents used for mineral, fatty acid, and amino acid analyses are described in the corresponding references.

### 2.4 Proximate composition

Proximate composition of the samples was determined in accordance with AOAC OMA^SM^ methods (28). Moisture (AOAC 925.09), ash (AOAC 923.03), and total dietary fiber (AOAC 985.29) were analyzed using the respective standard procedures. Crude protein was determined by the Kjeldahl method (AOAC 979.07, modified), applying nitrogen-to-protein conversion factors of *N* × 5.00 and *N* × 6.25, as appropriate. Total fat was quantified according to AOAC 920.85. Total carbohydrates were calculated by difference using the equation: %Total carbohydrates = 100 − (%Moisture + %Ash + %Protein + %Fat), while available carbohydrates were estimated as %Available carbohydrates = %Total carbohydrates − %Total dietary fiber. The %Protein considered for the calculation is the chitin-corrected protein content (Kp 6.25).

### 2.5 Insect exoskeleton chitin content

Chitin/Chitosan determination was performed by chemical hydrolysis (29) and posterior gravimetric analysis. Briefly, 3 g of sample was weighed onto 250 mL Erlenmeyer flask and 150 mL of HCl 1 mol L^−1^ solution were added, this mixture was agitated and incubated overnight on a water-bath at 97 °C. The mixture was then filtered and washed until the resulting solid residue was acid free. Said residue was transferred into a second Erlenmeyer flask and 150 mL NaOH 1 mol L^−1^ solution added and kept at 80 °C for 24 h. Finally, the mixture was filtered and washed with ultrapure water until base excess was removed. The residue obtained was freeze-dried and weighed (Precisa, Series 520, 0.1 mg readability, Moosmattstrasse, Dietikon Switzerland).

### 2.6 Vitamins and minerals

Mineral composition was determined according to AOAC OMA^SM^ methods; sodium, potassium, and magnesium were analyzed following AOAC 985.35; calcium and phosphorus according to AOAC 991.25; iron and copper by AOAC 999.11; and zinc by AOAC 969.32. Mineral content was determined using an atomic absorption spectrometer (200 Series, 280 FS AA, Agilent Technologies, Santa Clara, CA, USA) and spectrophotometer (PharmaSpec, UV-1700, Shimadzu Corporation, Kyoto Prefecture, Japan) see details in Cortés-Herrera et al. (30).

Meanwhile, for lipophilic vitamins analysis a previously homogenized subsample (2–3 g) weighed into a 50 mL conical centrifuge tube (CLS430829, polypropylene, Corning®, New York, USA) and 30 mL of chloroform containing BHT (0.1 g per 100 mL) was added. The mixture was vortexed for 1 min, followed by 20 min of orbital shaking at 350 rpm (Fermentation Desing Inc., Model 0S31, Allentown, PA, USA). The remnant suspension was centrifuged at 3,000 × g for 10 min; the organic phase was collected and transferred to a 125 mL Erlenmeyer flask. A second extraction was performed using an additional 30 mL of chloroform, and both organic phases were combined. Enough desiccant (Na_2_SO_4_) was added, and the mixture was shaken and filtered through Whatman filter paper (41, GE Healthcare, Chicago, IL, USA) into evaporation flasks. The solvent was evaporated at 40 °C (Multivapor P-6, Büchi, Flawil, Switzerland), and the residue was reconstituted in 2-propanol to a final volume of 5 mL. The solution was filtered through a 0.20 mm filter (syringe filters with PTFE hydrophobic membrane, catalog 5,191–5,912, Agilent Technologies, Santa Clara, CA, USA) into an HPLC vial (Agilent Technologies, Santa Clara, CA, USA) for analysis. In this case, vitamins were identified and quantified by HPLC-PDA by the following conditions. A 10 μL sample was injected onto a Supelcosil C_18_ column (4.6 × 150 mm, 3 μm, Supelco, Bellefonte, PA, USA). The mobile phase consisted of MeOH: Ultrapure water (95:5) in isocratic mode, with a flow rate of 1 mL min^−1^ at 35 °C for 70 min. Tocopherol peaks were identified at 295 nm and 285 nm using *α*-tocopherol acetate, α-tocopherol, *β*-tocopherol, *δ*-tocopherol, and *γ*-tocopherol standards, Retinol (acetate and palmitate), ergocalciferol and cholecalciferol peaks were identified at 325 nm and 264 nm, respectively. Quantification was performed using calibration curves with a coefficient of determination (R^2^) ≥ 0.995.

Moreover, hydrophilic vitamins analysis was performed using household methods based on Lykkesfeldt (31), Wechtersbach and Cigić (32), and Kim (33). For vitamin C quantification, a sample portion (approximately 3 g) was weighed into a 50 mL conical centrifuge tube (Corning^®^, USA) and 15 mL of metaphosphoric acid (MPA) at 40 g/100 g in ultrapure water. The mixture was vortexed for 2 min, followed by 1 h of orbital shaking at 350 rpm (Ilentown, PA, USA). Later, the mixture was centrifuged at 3,000 × *g* for 10 min at 4 °C; the supernatant was filtered and transferred to a 50 mL volumetric flask. A second extraction was performed using an additional 15 mL of MPA and diluted at 50 mL. A 2–3 mL of solution was filtered through a 0.45 mm filter Minisart^®^ RC25 (syringe filters with regenerated cellulose membrane, catalog 17765Q, Sartorius, Göttingen, Germany). Next, 500 μL of filtered solution was mixed with 500 μL tris [2-carboxyethyl] phosphine hydrochloride 40 mmol L^−1^ in ultrapure water in a ready-to-inject HPLC vial and kept at 30 °C for 40 min before injection. A Luna-C_18_ column (250 × 4.6 mm, 5 μm particle size; Phenomenex™, Torrence, CA, USA) was used to perform the separation, 20 μL was injected into the system in isocratic conditions using aqueous mobile phase at pH 2.6 (sulphuric acid, 2.81 mmolL^−1^ in ultrapure water) at a 0.8 mL min^−1^ flow, column temperature was kept at 30 °C and detection was performed at a wavelength of 245 nm. To assess the concentration, a five-point linear calibration curve was prepared by diluting the concentrated standard to appropriate volumes up to working concentrations ranging from 5 to 150 mg ascorbic acid L^−1^ in ultrapure water.

Finally, for B-complex vitamins analysis started with 2 g of defatted sample weighed into a 50 mL conical centrifuge tube (Corning^®^, USA) and 10 mL of HCl 0.10 molL^−1^, the mixture was vortexed for 15 min, followed by 1 h in a water-bath at 100 °C, then cooled and filtered to a 25 mL volumetric flask, final volume was make-up with HCl 0.10 molL^−1^. After that, 1 mL of solution and 1 mL of methanol was vortexed for 1 min in a 15 mL conical centrifuge tube (Corning^®^, USA) centrifuged at 3,000 × *g* for 10 min and pH 7 adjusted. Then 1 mL of solution was filtered through a 0.45 mm filter (Minisart^®^ RC25, Sartorius) in a ready-to-inject HPLC vial and 25 μL of the mixture was injected into the system. A Zorbax Eclipse Plus C_18_ column (250 mm × 4.6 mm, 5 μm particle size; Agilent Technologies, Santa Clara, Ca, USA) was used to perform the separation under gradient method using ultrapure water (solvent A) and MeOH (solvent B) both with 2.27 mmol L^−1^ heptanesulfonate salt at 0.5 mL min^−1^ was used. The mobile phase system was set as follows: starting at 10% B at 0 min, 10% B at 2 min, 70% B at 22 min, 70% B at 27 min, 10% B at 28 min, and 10% B at 35 min. Column temperature was kept at 40 °C. To assess the concentration, eight-point linear calibration curves were prepared by diluting the concentrated standard to appropriate volumes up to working concentrations range of 2–10 μg mL^−1^. Under these conditions wavelengths detection was 290 nm (pyridoxal 5′-phosphate hydrate and pyridoxine hydrochloride at 19.25 min), 270 nm (folic acid at 16.8 min, riboflavin at 18.5 min and thiamine hydrochloride at 23.2 min), 361 nm (cyanocobalamin at 16.2 min) and 260 nm (nicotinic acid at 7.6 min and niacinamide at 13.2 min).

All vitamins analysis was performed using a Shimadzu HPLC system (Shimadzu, Kyoto, Japan) equipped with a diode array detector (SPD-M20A), column oven (CTO-20A), autosampler (SIL-20A HT), and quaternary pump (LC-20AT).

Method performance was verified using the certified reference materials NIST 1849a Infant/Adult Nutritional Formula and NIST 1846 Infant Formula. Quality control materials included T18101QC Infant Formula for mineral analyses and T21124QC Powdered Baby Food for vitamin analyses. The laboratory operates under an ISO/IEC 17025 quality system and participates regularly in Fera FAPAS proficiency testing schemes. Recoveries ranged from 82 to 110% for vitamins, while mineral recoveries were greater than 80%, indicating acceptable analytical accuracy. Analytical precision was assessed through repeatability and reproducibility studies, and detailed reproducibility (RSDR) and HorRat values for mineral and vitamin determinations are provided in Supplementary Tables S1, S2, respectively. Limits of detection (mg/100 g) for minerals were 0.20 (Cu), 0.11 (Zn), 1.08 (Fe), 8.45 (P), 15.14 (K), 17.36 (Na), 3.61 (Mg), and 23.17 (Ca). For hydrophilic vitamins, limits of detection (mg/100 g) were 0.053 for pyridoxal 5′-phosphate and pyridoxine hydrochloride, 0.050 for folic acid and cyanocobalamin, 0.048 for riboflavin and thiamine hydrochloride, 0.19 for nicotinic acid, and 0.17 for niacinamide. Detection limits for retinol (acetate and palmitate), *δ*-tocopherol, and vitamin D (ergocalciferol and cholecalciferol) were 0.084, 0.073, and 0.13 mg/100 g, respectively. Compounds present below their respective detection limits were reported as not detected.

### 2.7 Amino acid profile and quality protein parameters

The amino acid profile was determined following the method described by Leiva et al. (34), in this case Infant/Adult nutritional formula NIST^®^ SRM^®^ 1849a amino acids was used for calibration. Essential amino acids (EAA) analyzed included histidine, leucine, phenylalanine, isoleucine, methionine, valine and lysine, whereas non-essential amino acids (NEAA) comprised arginine, glycine, glutamic acid, aspartic acid, proline, serine, cystine, and alanine. The analysis was performed at a flow of 2 mL min^−1^ using an Agilent Technologies LC system equipped high resolution column (150 × 4.6 mm, 3.5 μm, PN 963400–901260), a 1,260 infinity quaternary pump (61311C), column compartment (G1316A), an automatic liquid sampler module (ALS, G7129A) and a fluorescence detector (G1321A; Agilent Technologies, Santa Clara, CA, USA).

Protein quality was evaluated through the calculation of Amino Acid Score (AAS), Essential Amino Acid to Total Amino Acid Ratio (EAA/TAA), Essential Amino Acid Index (EAAI), predicted Biological Value (BV), and Protein Efficiency Ratio (PER_1-3_). However, because tryptophan and threonine were not individually quantified, they were not considered in any protein quality estimation; but tyrosine was only available as part of the combined phenylalanine + tyrosine value, some predictive indices should be interpreted as approximate estimations rather than fully comparable or definitive measures of protein biological quality. Thus, EAAI, BV, and PER_1-3_ values were included primarily for comparative nutritional interpretation and should be considered cautiously within the methodological limitations of the amino acid dataset.

The Amino Acid Score (AAS) was calculated using the FAO/WHO reference amino acid pattern for children aged 1–2 years–which represents the most stringent reference pattern for evaluation of dietary protein quality–according to the following equation:


$$\mathrm{AAS}=\frac{\mathrm{mg}\;\text{of amino acids in}\;1\;g\;\text{total protein}}{\mathrm{mg}\;\text{of amino acids in requirement pattern}}\times 100$$

The nutritional balance of amino acids was additionally assessed by calculating the ratio of total essential amino acids to total amino acids (EAA/TAA), which is commonly used as an indicator of protein nutritional quality.


$$\frac{\mathrm{EAA}}{\mathrm{TAA}}=\frac{\Sigma \mathrm{EAA}}{\Sigma \mathrm{TAA}}\times 100$$

The EAAI was calculated using whole egg protein as the reference standard following Amza et al. (35), according to the equation:


$$\text{EAAI}=\sqrt[{n}]{\frac{(\mathrm{Lys}\times \mathrm{Val}\times \mathrm{Met}\times \mathrm{Ile}\times \mathrm{Leu}\times \mathrm{Phe}\times \mathrm{His})a}{(\mathrm{Lys}\times \mathrm{Val}\times \mathrm{Met}\times \mathrm{Ile}\times \mathrm{Leu}\times \mathrm{Phe}\times \mathrm{His})b}}$$

where *n* represents the amino acids considered for the equation, *a* represents the content of each essential amino acid in the test sample and *b* represents the content of the corresponding amino acid in the reference protein (%).

The BV value was estimated according to Amza et al. (35) using the following relationship:


BV=1.09(EAAI)−11.7

The PER was estimated from the amino acid composition based on the regression equations proposed by Amza et al. (35). The PER values were calculated using the following equations:


$${\mathrm{PER}}_{1}=-0.684+0.456(\mathrm{Leu})-0.047(\mathrm{Pro})$$

$${\mathrm{PER}}_{2}=-0.468+0.454(\mathrm{Leu})-0.105(\mathrm{Tyr})$$

$$\begin{array}{l} {\mathrm{PER}}_{3}=-1.816+0.435(\mathrm{Met})+0.780(\mathrm{Leu})\\ +0.211(\mathrm{His})-0.944(\mathrm{Tyr}) \end{array}$$

### 2.8 Fatty acid profile and lipid quality indices

The fatty acid profile (saturated, monounsaturated, and polyunsaturated fatty acids) was determined following AOAC 996.06 and AOCS Ce 1e-91 using a GC/FID system model GC-2014 equipped with an AOC-20i automatic liquid sample injection system (Shimadzu Corporation, Nakagyo-ku, Kyoto, Japan; (36)). Based on the fatty acid composition, lipid nutritional quality was evaluated by calculating the Health Promoting Index (HPI), the Atherogenicity Index (AI), and the Thrombogenicity Index (TI), which are widely used indicators of the potential impact of dietary lipids on cardiovascular health.

The AI and TI were calculated according to the equations proposed by Ulbricht and Southgate (37), while the HPI was calculated as the reciprocal of AI, following previous literature (38). The indices were computed using the relative percentages of individual fatty acids as follows:

$$\mathrm{HPI}=\frac{\Sigma \text{MUFA}+\Sigma \text{PUFA}}{C12:0+(4\times C14:0)+C16:0}$$

$$\mathrm{TI}=\frac{C14:0+C16:0+C18:0}{0.5(\Sigma \text{MUFA})+0.5(\mathrm{\Sigma n}-6)+3(\mathrm{\Sigma n}-3)+(\frac{\mathrm{\Sigma n}-3}{\mathrm{\Sigma n}-6})}$$

$$\mathrm{AI}=\frac{C12:0+(4\times C14:0)+C16:0}{\Sigma \text{MUFA}+\Sigma \text{PUFA}}$$

where individual fatty acids are expressed as percentage of total identified fatty acids.

### 2.9 Statistical analysis

Nutritional characterization was performed using four independent biological replicates (*n* = 4), corresponding to four independently reared groups of *Gryllus* sp. Results are expressed as mean ± standard deviation (SD). No inferential statistical analyses were conducted, as the study focused on compositional characterization of a single species.

## 3 Results and discussion

### 3.1 Insect identification and morphological description

**Figure 1:**
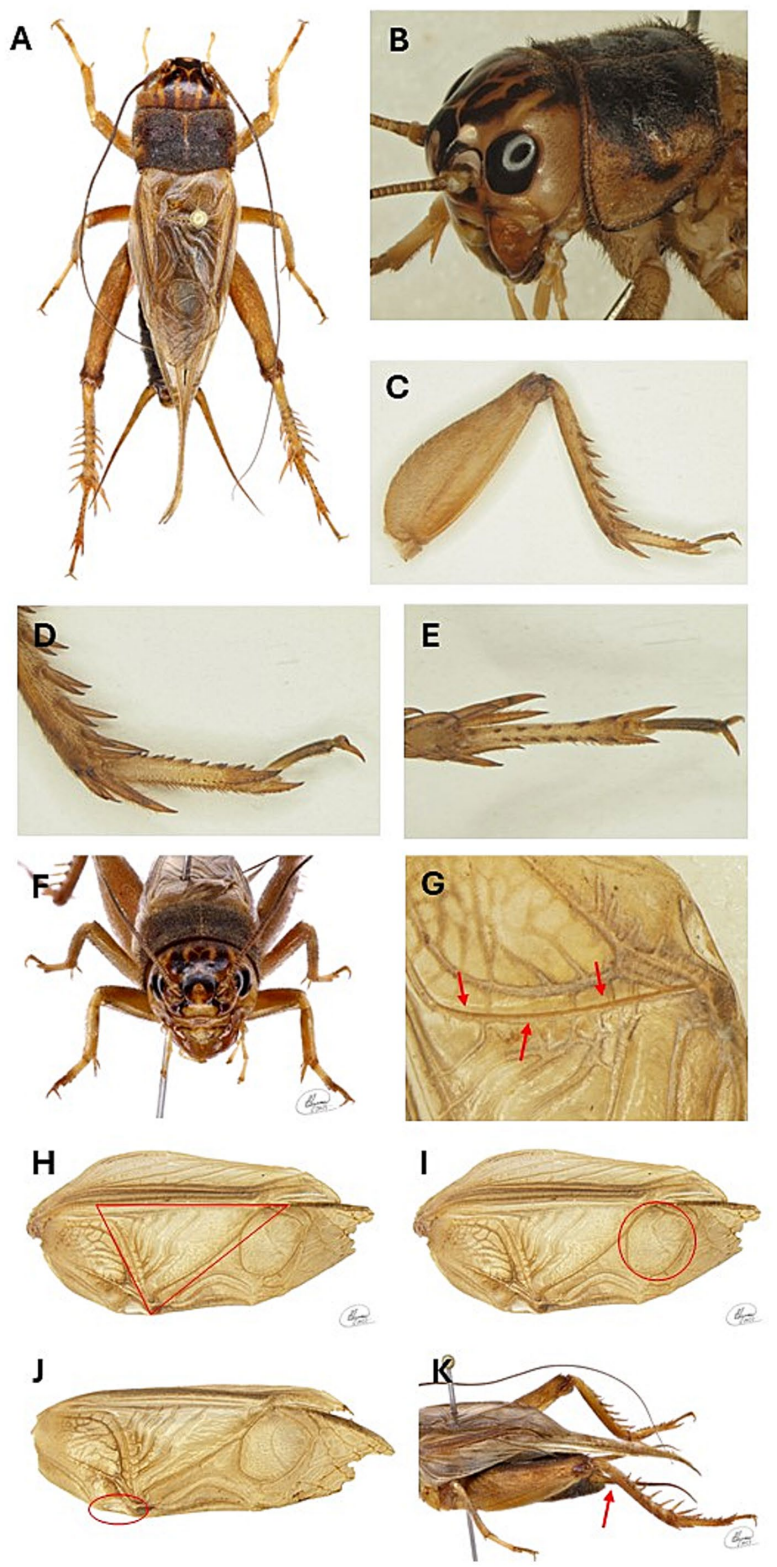
External morphology of *Gryllus* sp. examined in this study. **(A)** Habitus, dorsal view. **(B)** Pronotum, dorsal-lateral view. **(C)** Hind leg. **(D, E)** Hind tibia and tarsus showing dorsal spines and apical spurs. (**F**) Head, superior-frontal view. **(G)** Stridulatory file on the right tegmen. **(H)** Harp of the tegmen. **(I)** Mirror of the tegmen. **(J)** Scraper region in the tegmina. **(K)** Male subgenital plate, dorsal view. Images courtesy of Humberto J. Lezama, Museo de Insectos, CIPROC, Escuela de Agronomía, Universidad de Costa Rica. Reproduced with permission.

Specimens were taxonomically assessed based on external morphology following standard diagnostic characters for Gryllidae and Gryllinae (39, 40). Individuals exhibited a robust, medium-to-large body size and a subcylindrical habitus typical of field crickets (Figure 1A). The pronotum was broad, transverse, and dorsally smooth to finely pubescent, without marked lateral expansions (Figure 1B). Hind femora were strongly developed, and the hind tibiae bore multiple dorsal spines and well-developed apical spurs (Figures 1C–E). Antennae were long and filiform, and the head was broad with large compound eyes (Figure 1F).

Specimens were assigned to the genus *Gryllus* based on external morphology and tegminal characters (39, 40). Males exhibit well-developed forewings with a typical stridulatory apparatus, including a distinct file with regularly spaced teeth (Figure 1G), a well-defined harp (Figure 1H), and a large suboval mirror (Figure 1I), all consistent with diagnostic features of *Gryllus* within Gryllinae. The general tegminal venation pattern, the presence of scrapper, and the relative development of the dorsal field further support this placement (Figure 1J). Males also possess a long pair of cerci approximately as long as the half of the abdomen (Figure 1K), a condition consistent with species of *Gryllus*. The combination of a robust body, transverse pronotum, characteristic tegminal venation, and cerci morphology supports placement within *Gryllus*.

The species of cricket studied belongs to the genus *Gryllus* and is morphologically distinct from other species for human consumption such as *G. bimaculatus*, *G. assimilis* and *G. testaceus*, may correspond to an undescribed or currently unresolved member of the *Gryllus* field cricket complex, probably *G. locorojo* or a similar species (Dr. David Weissman, personal communication; (40)); however, species-level identification requires molecular, acoustic, and genitalia-based evidence. Despite the clear generic placement, species-level identification was not attempted because molecular confirmation was not performed, which are typically required for reliable species delimitation in *Gryllus* (39, 40). Therefore, the material is conservatively referred to as *Gryllus* sp. for the purposes of the present nutritional and anatomical characterization.

Additionally, morphological comparison indicates that the examined material belongs to the *Gryllus* field cricket complex and differs from commonly farmed or reported edible cricket species. In particular, the overall body robustness (Figure 1A), pronotal proportions (Figure 1B), and tegminal configuration (Figures 1A,G–J) distinguish the specimens from typical *Acheta* species (e.g., *Acheta domesticus*), which generally exhibit a slenderer habitus and a different tegminal venation pattern (39). The absence of ovipositor indicates that this individual is a male (Figure 1K).

Likewise, the relatively large body size, well-developed ovipositor (not shown images), and tegminal morphology are inconsistent with smaller-bodied genera such as *Gryllodes*. Within *Gryllus*, the specimens show general affinity with the Neotropical *Gryllus assimilis* species group, which includes several morphologically similar taxa (40). Nevertheless, reliable separation among species of this complex requires detailed examination of male genitalia, analysing calling songs through sonograms, and/or molecular markers. Consequently, although the material may be related to taxa such as *G. assimilis*, *G. bimaculatus*, or allied Neotropical species, the available morphological evidence supports only a conservative identification as *Gryllus* sp. pending dedicated taxonomic revision.

### 3.2 Proximate composition

The chemical composition of *Gryllus* sp. was evaluated not only to determine its nutritional quality but also to explore its potential contribution to species-level biochemical characterization. The proximate composition of *Gryllus* sp. powder (Table 1) is largely consistent with previously published data for other *Gryllus* species. Nonetheless, variability across studies remains evident and likely reflects differences in species or strain, rearing substrates, developmental stage, processing conditions, and analytical methodologies.

**Table 1:** Proximate composition of *Gryllus* sp. powder.

Proximate composition^1^	*Gryllus* sp.	
Concentration^1^, g/100 g DM
Moisture	6.48 ± 2.36
Ash	5.01 ± 0.16
Dietary fiber	7.43 ± 0.24
Chitin	7.15 ± 0.41
Protein^2^	
Crude protein (*N_T_* × *K*_*p* 5.00_)	51.19 ± 3.09
Crude protein (*N*_chitin corrected_ × *K*_*p* 6.25_)	61.48 ± 3.84
Total fat	11.65 ± 0.64
Saturated fat	3.78 ± 1.14
Unsaturated fat	4.73 ± 1.39
Polyunsaturated fat	3.14 ± 2.23
Total carbohydrates	15.39 ± 1.85
Available carbohydrates	7.96 ± 2.07

^1^All values are expressed as mean ± standard deviation. ^2^NT: total nitrogen (10.24 g/100 g); Kp 5.00 reported for *Gryllus bimaculatus* by Ritvanen et al. (41); *N*_chitin corrected_: *N*_*T*_-Nitrogen in chitin (9.84 g/100 g).

The crude protein content calculated using a nitrogen-to-protein conversion factor (Kp) of 5.00 (51.19 g/100 g DM) aligns with the recommendation proposed for *G. bimaculatus* by Ritvanen et al. (41), which accounts for the contribution of non-protein nitrogen associated with chitin. However, the direct application of a species-specific Kp determined for *G. bimaculatus* may not be fully appropriate for a distinct *Gryllus* species, as nitrogen-to-protein ratios can vary among species depending on amino acid composition and chitin content. In the absence of a validated species-specific conversion factor, correction of total nitrogen for chitin prior to applying the conventional Kp of 6.25 represents a more robust and chemically justified approach. Under this correction, protein content reached 61.48 g/100 g DM, a value comparable to those reported for *G. bimaculatus* by Murugu et al. (42), Peh et al. (43), Taufek et al. (44), and Udomsil et al. (45), who reported protein contents ranging from approximately 57–61 g/100 g. According to this, the chitin-corrected value is considered the most representative estimate of true protein content in the present material. These findings expand that reliance on a universal Kp of 6.25 without nitrogen correction may lead to overestimation of protein in insects, while extrapolation of species-specific Kp values across taxa may introduce additional bias.

Ash content of 5.01 g/100 g DM was comparable to values reported by Murugu et al. (42) and Peh et al. (43), and within the range observed for other *Gryllus* species. Total fat content, 11.65 g/100 g DM, was lower than values reported by Jayanegara et al. (46), Peh et al. (43) and Udomsil et al. (45), but like that reported by Taufek et al. (44), underscoring the strong influence of diet composition and lipid extraction procedures. Dietary fiber and chitin contents were also within the ranges described for *Gryllus* species, including *G. testaceus* (47) and *G. assimilis* (46).

Taken together, the proximate profile of *Gryllus* sp. falls within the compositional ranges reported for *G. bimaculatus*, *G. testaceus*, and *G. assimilis*, indicating broad nutritional similarity within the genus. However, the overlap in proximate composition among these species prevents definitive taxonomic identification based solely on macronutrient data. While the compositional patterns support classification within the genus *Gryllus*, they do not provide sufficient discriminatory resolution to conclude that the analyzed material corresponds to any of the three previously characterized species. Instead, the observed differences should be interpreted as evidence of nutritional variability among *Gryllus* populations. Because nutritional composition is influenced by multiple biological and environmental factors, it should not be used as evidence for species delimitation. Although nutritional composition alone cannot support species delimitation, the consistent compositional differences observed across *Gryllus* taxa suggest that biochemical and nutritional profiling may provide complementary information for comparative characterization when interpreted alongside morphological, ecological, and molecular evidence. Overall, these results should be interpreted cautiously as indicators of compositional variability within the genus rather than as independent evidence of species-level differentiation.

### 3.3 Micronutrient profile

The mineral and vitamin composition of *Gryllus* sp. in the present study (Table 2) differed substantially from values reported for other *Gryllus* species. When compared with *Gryllus bimaculatus* (42, 45), calcium (313.75 mg/100 g DM), potassium (1,037.50 mg/100 g DM), magnesium (129.50 mg/100 g DM), phosphorus (915.00 mg/100 g DM), and sodium (387.00 mg/100 g DM) were consistently higher. For instance, Udomsil et al. (45) reported Ca 105.14, K 321.71, Mg 72.94, P 702.02, and Na 88.84 mg/100 g DM, while Murugu et al. (42) reported Ca 72.70, K 39.54, Mg 29.13, and Na 166.50 mg/100 g DM. In contrast, iron and zinc were comparable to values reported by Udomsil et al. (45), Fe 7.16 and Zn 14.39 mg/100 g DM but lower than those described by Murugu et al. (42) (Fe 12.33 and Zn 23.74 mg/100 g DM). Copper concentration (1.28 mg/100 g DM) was lower than in both comparative studies. Overall, while trace mineral levels fall within previously reported ranges for *G. bimaculatus*, macro mineral concentrations, particularly potassium, calcium, and magnesium, were substantially higher for *Gryllus* sp. in the present work.

**Table 2:** Vitamin and mineral profile of *Gryllus* sp. powder.

Vitamin and mineral^1^	*Gryllus* sp.
	Concentration (mg/100 g DM)
Sodium (Na)	387.00 ± 54.03
Calcium (Ca)	313.75 ± 42.26
Potassium (K)	1,037.50 ± 78.90
Iron (Fe)	6.68 ± 0.88
Magnesium (Mg)	129.50 ± 5.80
Phosphorus (P)	915.00 ± 12.91
Copper (Cu)	1.28 ± 0.16
Zinc (Zn)	13.47 ± 0.56
α-tocopherol acetate	5.94 ± 0.47
γ- and β-tocopherol	8.83 ± 0.58
α-tocopherol	23.80 ± 2.93
Vitamin C	11.64 ± 1.80
	Concentration (μg/100 g DM)
Riboflavin	70.43 ± 3.46
Thiamine hydrochloride	16.44 ± 14.02
Folic acid	243.50 ± 118.94

^1^All values are expressed as mean ± standard deviation. Only vitamins above limit detection were included.

Comparison with adult *Gryllus assimilis* reported by Oliveira et al. (48) also revealed distinct mineral differences. While magnesium levels were comparable between studies, the present *Gryllus* sp. showed higher concentrations of calcium, potassium, phosphorus, and sodium. In contrast, iron, zinc, and copper were lower than those reported for *G. assimilis*. Overall, the mineral profile of this *Gryllus* sp. does not fully correspond to either *G. bimaculatus* or *G. assimilis*.

Vitamin composition also showed marked variability across species. *α*-tocopherol (23.80 mg/100 g DM) in the present study was substantially lower than 1,255 mg/100 g DM reported for *G. bimaculatus* by Murugu et al. (42), yet markedly higher than the value reported for adult *G. assimilis* (0.98 mg/100 g) by Oliveira et al. (48). Vitamin C (11.64 mg/100 g DM) greatly exceeded the concentration reported for adult *G. assimilis* (0.94 mg/100 g). Riboflavin (70.43 μg/100 g DM) was quantified in the present work but reported as not detected in *G. assimilis* (48). Thiamine and folate were lower than those reported for *G. bimaculatus* (42). The combined mineral and vitamin patterns therefore reveal a distinct micronutrient signature when compared across multiple *Gryllus* species. In case of retinol (acetate and palmitate), *δ*-tocopherol and vitamin D (ergocalciferol and cholecalciferol) were under detection limits (0.084 mg/100 g; 0.073 mg/100 g and 0.13 mg/100 g, respectively).

The magnitude and pattern of variation observed across studies likely reflect differences in feed composition, mineral supplementation, environmental rearing conditions, developmental stage, post-harvest processing, and analytical methodologies (42, 45, 48). Insects are known to bioaccumulate dietary minerals, and micronutrient profiles are highly responsive to substrate composition. Consequently, direct comparisons among studies should be interpreted with caution because the observed differences may arise from both biological and methodological factors.

The reported concentrations of several macro minerals in the present study, particularly calcium, potassium, phosphorus, and sodium, together with the observed vitamin profile, contribute valuable new information to the growing body of knowledge on the nutritional composition of *Gryllus* sp. These findings demonstrate the broad range of micronutrient profiles that can occur within Gryllus populations and production systems. These findings expand the available nutritional information for *Gryllus* sp. and illustrate the variability that may occur among edible cricket populations. Future studies conducted under standardized rearing conditions and harmonized analytical methodologies, ideally combined with molecular species identification, would help clarify the relative contributions of genetic and environmental factors to micronutrient composition among *Gryllus* populations.

### 3.4 Amino acid profile and protein nutritional quality

The essential amino acid (EAA) profile of the analyzed sample (*Gryllus* sp.) indicated a generally favorable amino acid balance when expressed as mg AA per g protein and evaluated against FAO/WHO reference patterns for adults and children aged 1–2 years (Table 3). Although, comparison with literature data for *G. bimaculatus*, *G. testaceus*, and *G. assimilis* revealed clear quantitative differences in amino acid distribution.

**Table 3:** Amino acid composition (mg AA/g protein) of *Gryllus* sp. compared with reference amino acid values for *Gryllus bimaculatus*, *Gryllus testaceus* and *Gryllus assimilis*, and the WHO amino acid reference patterns for adults and children (1–2 years).

Amino acid composition	*Gryllus* sp.^1^	*G. bimaculatus* ^2^	*G. testaceus* ^3^	*G. assimilis* ^4^	WHO^5^ adults	WHO^5^ 1–2 years
EAA (mg/g protein)						
Histidine	115.3 ± 42.5	25.9	33.3	110.9	15.0	18.0
Isoleucine	48.5 ± 11.7	38.7	53.0	45.3	30.0	31.0
Leucine	58.6 ± 11.9	63.9	94.5	74.9	59.0	63.0
Lysine	34.8 ± 19.9*	47.6	82.2	65.9	45.0	52.0
Methionine + Cystine	68.5 ± 51.8	20.4*	50.4	18.8*	22.0	26.0
Phenylalanine + Tyrosine	48.5 ± 12.7	82.5	116.6	101.9	38.0	46.0
Threonine	NR	27.5	47.2	46.1	23.0	27.0
Tryptophan	NR	4.4	NR	NR	6.0	7.4
Valine	45.7 ± 17.3	57.7	75.8	62.8	39.0	42.0
NEAA (mg/g protein)						
Arginine	103.1 ± 37.6	57.2	63.1	69.0	–	–
Aspartic acid (+Asn)	71.3 ± 23.1	47.3	107.9	88.0	–	–
Glutamic acid (+Gln)	82.2 ± 21.1	111.5	155.6	130.0	–	–
Serine	33.5 ± 2.5	54.5	63.8	41.4	–	–
Glycine	70.4 ± 8.8	54.5	62.1	63.6	–	–
Alanine	5.6 ± 0.9	77.3	95.2	81.3	–	–
Proline	34.2 ± 21.0	46.3	77.2	NR	–	–
Protein quality parameters						
EAA/TAA (%)	56.51	36.9 32.85	42.0 37.79	48.6 43.77	–	–
EAAI (%)	111.08	60.4 72.16	109.3109.28	104.4104.68	–	–
AAS (%)	66.88	54.5 54.49	106.6106.64	72.3 72.31	–	–
BV	50.0 49.87	28.3 24.08	34.1 29.46	41.2 35.98	–	–
PER_1_	1.83	2.01	3.27	2.73	–	–
PER_2_	2.19	1.95	3.12	2.29	–	–
PER_3_	8.17	0.2	1.33	1.43	–	–

^1^Values are expressed as mean ± standard deviation. Tryptophan, threonine, and tyrosine were not included. AA, amino acid; EAA, essential amino acids; NEAA, non-essential amino acids; EAA/TAA, essential amino acid on total amino acid; EAAI, essential amino acid index; BV, biological value; PER, protein efficiency ratio; NR, not reported. Data sources: ^2^ Udomsil et al. (45); ^3^ Wang et al. (47); ^4^ Jayanegara et al. (46); ^5^ FAO/WHO/UNU (55). *Limiting AA. Tyrosine was not individually quantified and was only available as part of the combined phenylalanine + tyrosine value; therefore, predictive indices requiring individual Tyr values were interpreted cautiously.

Regarding branched-chain amino acids, isoleucine (Ile) and valine (Val) in the present *Gryllus* sp. sample exceeded FAO requirements for both age groups and fell within the lower-to-intermediate range reported for the reference species. Leucine (Leu = 58.64 mg/g protein) was comparable to values reported for *G. bimaculatus* but notably lower than those described for *G. testaceus* and *G. assimilis*. Although leucine approached the adult FAO requirement (59 mg/g protein), it did not fully meet the requirement for children, indicating borderline adequacy for younger age groups.

The most pronounced divergence relative to the reference species was observed for lysine (Lys). Accordingly, Lys was identified as the first limiting amino acid based on the calculated amino acid score (AAS). The mean Lys concentration in the examined *Gryllus* sp. sample was lower than values reported for *G. bimaculatus* (45), *G. testaceus* (47), and *G. assimilis* (46), and fell below the FAO/WHO reference patterns for both adults (45 mg/g protein) and children (52 mg/g protein). However, the relatively large standard deviations observed for Lys and some other amino acids, including histidine and methionine + cystine, indicate considerable variability among replicates and thus these findings should be interpreted cautiously. Such variability may reflect biological heterogeneity, analytical variation, or differences associated with sample preparation and amino acid quantification.

When the literature values for *G. bimaculatus*, *G. testaceus*, and *G. assimilis* (45–47) are directly compared with WHO reference patterns for adults and children (1–2 years), clear differences in essential amino acid adequacy are observed. In *G. bimaculatus*, sulfur amino acids (Met + Cys; 20.4 mg/g protein) are below the WHO requirements for both age groups, and Trp (4.4 mg/g protein) is also lower than the reference values. Similarly, in *G. assimilis*, Met + Cys (18.8 mg/g protein) fall below WHO recommendations. In contrast, *G. testaceus* meets or exceeds the WHO reference values for the essential amino acids reported in the dataset. Across all three species, His and aromatic amino acids (Phe + Tyr) are present at concentrations well above the WHO reference patterns.

Alanine (Ala) values (5.59 mg/g protein) observed in the present study were notably lower than those reported for comparative *Gryllus* species (77.3–95.2 mg/g protein). Although the analytical procedures and calculations were rechecked, this difference may reflect methodological variability, matrix effects, or biological differences associated with the analyzed population and therefore should be interpreted cautiously.

Amino acid profiling provides useful nutritional information for comparisons among studies, although it should not be interpreted as evidence of taxonomic differentiation because amino acid composition is also influenced by environmental and production factors. The variability associated with rearing substrate, developmental stage, processing conditions, nitrogen-to-protein conversion factors, and analytical methodologies must be carefully considered. Therefore, while the observed Lys deficit and amino acid scoring pattern highlight meaningful nutritional differences, definitive taxonomic interpretation requires integration with morphological and molecular evidence. Standardized analytical approaches and harmonized reporting formats are essential to strengthen interspecies of nutritional comparisons within the genus *Gryllus*.

Beyond its compositional characterization, *Gryllus* sp. powder may have potential applications as a sustainable ingredient for human nutrition and functional food development. Due to its high protein content, favorable essential amino acid contribution, mineral content, and unsaturated fatty acid profile, it could be incorporated into protein-enriched flours, bakery products, snacks, pasta, and hybrid formulations combining insect and plant proteins. However, the present nutritional evaluation should not be interpreted as support for exclusive dietary reliance on cricket-derived products, since nutritionally balanced diets require dietary diversity and complementary nutrient sources. In this context, the relatively low lysine content identified in the present work could be nutritionally compensated through formulation strategies involving lysine-rich ingredients such as legumes, nuts, or oilseed-based products. Additionally, previous studies have demonstrated that the nutritional composition of edible insects may be influenced by rearing conditions and feeding substrates (49, 50), suggesting that controlled production systems could potentially improve specific nutritional attributes, including amino acid and fatty acid composition. Overall, *Gryllus* sp. may represent a promising complementary protein ingredient within sustainable and diversified food systems rather than a standalone nutritional source.

Protein quality indicators revealed interspecific variability among the evaluated *Gryllus* species (Table 3). The EAA/TAA ratio of this *Gryllus* sp. (56.51%) was higher than that reported for *G. bimaculatus* (32.85%) and *G. testaceus* (37.79%), and comparable to *G. assimilis* (43.77%), confirming that the overall essential amino acid balance of the analyzed material falls within the upper range reported for the genus. Values above 40% are generally considered indicative of proteins with good nutritional quality, supporting the potential of *Gryllus* spp. as valuable alternative protein sources. However, these indices should be interpreted cautiously because some amino acids required for their complete calculation, particularly tryptophan, threonine, and individual tyrosine values, were not experimentally quantified. Consequently, these parameters are presented primarily as approximate comparative nutritional indicators rather than definitive measures of protein biological quality or digestibility. Despite these limitations, the overall amino acid balance and EAA/TAA ratio support the potential nutritional relevance of *Gryllus* sp. as an alternative protein source.

In contrast, the essential amino acid index (EAAI) showed a different ranking pattern. The studied *Gryllus* sp. exhibited an intermediate EAAI (111.08%), higher than *G. bimaculatus* (72.16%) and *G. assimilis* (109.28%), but lower than *G. testaceus* (104.68%). This divergence reflects differences in amino acid proportionality relative to the egg reference protein and highlights that EAA/TAA and EAAI capture complementary aspects of protein quality. Although EAAI values above 100% suggest a highly favorable essential amino acid balance relative to the egg reference protein, this index should be interpreted alongside limiting amino acid scores and digestibility-related metrics.

The predicted biological value (BV) followed a similar trend, with *Gryllus* sp. (49.87) exceeding *G. bimaculatus* (24.08) and approaching *G. testaceus* (29.46), although remaining lower than *G. assimilis* (35.98). Regarding these values, all *Gryllus* showed a limited to moderate protein BV. The predicted BV provides an estimate of the potential nitrogen retention based on essential amino acid composition; nevertheless, it does not account for protein digestibility or bioavailability.

Protein efficiency ratio (PER) estimates further emphasized species-dependent variation. The PER₁ and PER₂ values of the present *Gryllus* sp. were moderate (1.83 and 2.19, respectively), lower than those of *G. testaceus* but generally comparable to or higher than *G. bimaculatus*. However, wider dispersion was observed among PER predictive equations, particularly PER₃, which yielded very high values for *Gryllus* sp., overscoring the known limitations of regression based PER estimations when applied across diverse insect matrices.

Collectively, these results confirm that although members of the genus *Gryllus* consistently exhibit favorable protein quality, meaningful quantitative differences exist among species. Such variability likely reflects the combined influence of genetic background, feeding substrate, developmental stage, and methodological factors. From a nutritional application perspective, the relatively high EAA/TAA ratio but Lys limitation observed in this *Gryllus* sp. suggests that targeted amino acid complementation—particularly with Lys-rich ingredients such as legumes and nuts—could further enhance its suitability for human food formulations.

### 3.5 Fatty acids profile and lipid quality indices

The fatty acid profile of *Gryllus* sp. was dominated by unsaturated fatty acids, with linoleic acid (C18:2 n–6; 34.29 g/100 g lipids) and oleic acid (C18:1; 30.66 g/100 g lipids) as the most abundant components (Table 4). This predominance of unsaturated C18 fatty acids contributes to a favorable lipid composition from a nutritional perspective, particularly due to the relatively high proportion of polyunsaturated and monounsaturated fatty acids compared with saturated fatty acids. The sample also contained measurable amounts of *α*-linolenic acid (C18:3 n–3; 3.77 g/100 g lipids) and EPA (C20:5 n–3; 0.50 g/100 g lipids), indicating the presence of nutritionally relevant n–3 fatty acids, although the overall profile remained predominantly n–6-driven.

**Table 4:** Fatty acids profile (g/100 g lipids) and cardiometabolic indicators of *Gryllus* sp. powder.

Fatty acid profile	Fatty acid content (g/100 g lipids)
Lauric acid (C_12:0_)	0.01 ± 0.00
Myristic acid (C_14:0_, tetradecanoic acid)	0.05 ± 0.01
Pentadecanoic acid (C_15:0_)	0.57 ± 0.10
Palmitic acid (C_16:0_)	11.27 ± 1.92
Palmitoleic acid (C_16:1_)	1.39 ± 0.24
Margaric acid (C_17:0_)	0.06 ± 0.01
Margaroleic acid (C_17:1_)	0.30 ± 0.05
Stearic acid (C_18:0_, octadecanoic acid)	12.41 ± 2.11
Oleic acid	30.66 ± 5.21
11,14-Eicosadienoic acid, methyl ester	0.41 ± 0.07
Linoleic acid (C_18:2_, n-6)	34.29 ± 5.83
Gamma-linolenic acid, methyl ester	0.06 ± 0.01
10-Nonadecenoic acid, methyl ester	0.04 ± 0.01
Alfa-linolenic acid (C_18:3_, ALA, n-3)	3.77 ± 0.64
Araquidic acid (C_20:0_)	4.19 ± 0.71
8,11,14-Eicosatrienoic acid, methyl ester	0.15 ± 0.03
Behenic acid (C_22:0_)	0.34 ± 0.06
5,8,11,14,17-Eicosapentaenoic acid, methyl ester, (all-Z)-	0.50 ± 0.09
Lignoceric acid (C_24:0_)	0.07 ± 0.01
Grouped lipids (g/100 g lipids)	
Saturated (SFA)	28.72 ± 2.73
Monounsaturated (MUFA)	32.10 ± 2.46
Polyunsaturated (PUFA)	39.19 ± 3.74
Trans fats	0.00 ± 0.00
Cardiometabolic indicators	
Health-Promoting Index (HPI)	6.21
Atherogenicity Index (AI)	0.63
Thrombogenicity Index (IT)	0.53

Among saturated fatty acids, stearic acid (C18:0; 12.41 g/100 g lipids) and palmitic acid (C16:0; 11.27 g/100 g lipids) were the most abundant SFAs, whereas short- and medium-chain SFAs associated with higher atherogenic potential (C12:0 and C14:0) were detected only at very low concentrations. Overall, grouped fatty acids followed the distribution PUFA (39.19%) > MUFA (32.10%) > SFA (28.72%), with no detectable trans fatty acids. Consistent with this composition, Gryllus sp. exhibited relatively low atherogenicity (AI = 0.63) and thrombogenicity (TI = 0.53) indices, together with a comparatively high health-promoting index (HPI = 6.21), suggesting a potentially favorable cardiometabolic lipid profile from a compositional standpoint (51, 52). When benchmarked against conventional animal meats, the lipid indices of *Gryllus* sp. compare favourably, particularly for thrombogenicity. For example, a comprehensive report summarizing meat lipid indices indicates poultry AI = 0.37–0.45 and TI = 0.81–0.87, pork AI = 0.50–0.53 and TI = 1.09–1.18, and beef AI = 0.93 and TI = 1.93 (53). Under this context, the TI of *Gryllus* sp. (0.53) is markedly lower than values commonly reported for beef, pork, and even poultry, suggesting a comparatively reduced thrombogenic potential of its lipid fraction. Meanwhile, the AI (0.63) is within the range often reported for pork and some beef datasets, though higher than typical poultry values in that reference, indicating that the atherogenic component—driven largely by C16:0 and C14:0—remains relevant despite the high PUFA and MUFA content (53, 54).

## 4 Conclusion

The present study provides a comprehensive nutritional characterization of *Gryllus* sp., highlighting its high protein content, favorable unsaturated fatty acid profile, relevant mineral composition, and potentially valuable amino acid contribution for human nutrition. The results support the potential use of *Gryllus* sp. as a complementary ingredient in sustainable food formulations and alternative protein systems. Despite this, some nutritional limitations, particularly regarding lysine content, indicate that its optimal use may require combination with other complementary protein sources. The comparative nutritional differences observed between *Gryllus* sp. and related cricket species suggest that biochemical composition may provide complementary information for species characterization when interpreted together with morphological and molecular approaches. Nevertheless, these findings should not be considered sufficient for taxonomic differentiation on their own. Future studies should include molecular species confirmation, evaluation of rearing substrate effects on nutritional composition, digestibility and bioavailability analyses, and the development of food formulations incorporating *Gryllus* sp. powder into functional or protein-enriched products.

## Data Availability

The original contributions presented in the study are included in the article/Supplementary material, further inquiries can be directed to the corresponding authors.
