# Supplementary material for: Chemical characterization and nutritional potential of a Costa Rican edible cricket (Gryllus sp.) for human consumption
Source: Front Nutr. 2026 Jul 29;13:1816151. doi: 10.3389/fnut.2026.1816151 (PMC13463703; doi:10.3389/fnut.2026.1816151)
Supplement: Supplementary file 1 [file Table_1.docx]

**Supplementary material**

**Supplementary Table S1. Reproducibility (RSDR) and HorRat values for mineral determinations used in method validation and proficiency testing.**

| **Analyte** | **RSDR* (%)** | **HorRat** |
| --- | --- | --- |
| Calcium (mg/100 g) | 5.25–17.77 | 1.20–2.12 |
| Copper (mg/100 g) | 4.52–13.33 | 0.34–0.95 |
| Iron (mg/100 g) | 8.01–12.52 | 0.92–1.28 |
| Potassium (mg/100 g) | 2.17–8.65 | 0.36–1.68 |
| Magnesium (mg/100 g) | 0.58–8.76 | 0.09–1.13 |
| Sodium (mg/100 g) | 2.95–8.39 | 0.31–1.94 |
| Zinc (mg/100 g) | 3.35–17.06 | 0.36–1.91 |
| Phosphorus (mg/100 g) | 1.89-7.38 | 0.37-1.43 |

*RSDR, relative standard deviation of reproducibility. Values were obtained from method validation and proficiency testing performed across different matrices under the laboratory's ISO/IEC 17025 quality system.

**Supplementary Table S2. Reproducibility (RSDR) and HorRat values for vitamin determinations used in method validation and proficiency testing.**

| **Analyte** | **RSDR* (%)** | **HorRat** |
| --- | --- | --- |
| Vitamins A and E (mg/kg) | 8.80 | 1.36 |
| Vitamins D and K (mg/kg) | 16.25 | 1.47 |
| Vitamin C (mg/100 g) | 8.27 | 1.15 |
| Thiamine (mg/100 g) | 23.47 | 1.94 |
| Riboflavin (mg/100 g) | 12.82 | 1.19 |
| Vitamin B12 (µg/kg) | 11.33 | 0.28 |
| Folic acid (µg/100 g) | 19.19 | 1.32 |
| Nicotinic acid (mg/100 g) | 19.18 | 1.91 |
| Niacinamide (mg/100 g) | 11.11 | 1.45 |
| Pyridoxine (mg/100 g) | 4.90 | 0.45 |

*RSDR, relative standard deviation of reproducibility. Values were obtained from FAPAS quality control materials and proficiency testing schemes. HorRat values between approximately 0.5 and 2.0 are generally considered indicative of satisfactory analytical performance.
